# Severe *GBA1* variants drive the *GBA1*-PD clinical phenotype: implications for counselling and clinical trials

**DOI:** 10.1038/s41531-025-01063-3

**Published:** 2025-10-01

**Authors:** Elisa Menozzi, Sara Lucas Del Pozo, Jane Macnaughtan, Roxana Mezabrovschi, Sofia Koletsi, Pierfrancesco Mitrotti, Luca Gallo, Rosaria Calabrese, Marco Toffoli, Nadine Loefflad, Franco Valzania, Francesco Cavallieri, Valentina Fioravanti, Selen Yalkic, Naomi Limbachiya, Fabio Blandini, Micol Avenali, Anthony HV Schapira

**Affiliations:** 1 Department of Clinical and Movement Neurosciences, Queen Square Institute of Neurology, University College London (UCL), London, UK; 2 Aligning Science Across Parkinson’s (ASAP) Collaborative Research Network, Chevy Chase, MD 20815 USA; 3 Liver Failure Group, Institute for Liver and Digestive Health, University College London, London, UK; 4 Department of Brain and Behavioral Sciences, University of Pavia, Pavia, Italy; 5 IRCCS Mondino Foundation, Pavia, Italy; 6 Neurology Unit, Neuromotor and Rehabilitation Department, Azienda USL-IRCCS di Reggio Emilia, Reggio Emilia, Italy; 7 Ca’ Granda IRCCS Foundation, Ospedale Maggiore Policlinico, Milan, Italy

**Keywords:** Parkinson's disease, Risk factors, Mutation

## Abstract

Variants in the *GBA1* gene are the commonest genetic risk factor for Parkinson disease (PD). Genotype-phenotype correlations exist but with conflicting data. Here, we compared the clinical phenotype of 183 idiopathic PD (iPD) patients, 39 severe *GBA1*-PD, 24 mild *GBA1*-PD, and 55 risk *GBA1*-PD. Compared to iPD, we observed that only severe *GBA1*-PD patients had a distinctive, more several clinical profile, characterised by worse depression, hyposmia, cognitive dysfunction, and possibly constipation.

## Introduction

Variants in the *GBA1* gene are highly prevalent in the Parkinson disease (PD) population (~10–15%)^1,2^. In recent years, the increased recourse to *GBA1* genotyping in PD^3^, the different response of patients carrying *GBA1* variants (*GBA1*-PD) to advanced treatments for PD (i.e., deep brain stimulation-DBS)^4^, and the growing number of clinical trials testing *GBA1*-targeted therapies^5^, have created the need for standardized guidelines for genetic counselling, management and selection of patients for clinical trials^1^.

Major challenges in *GBA1*-PD include the differences in disease penetrance, and clinical severity and progression according to *GBA1* variant type^5^. The odds ratios for PD development are higher for severe *GBA1* variants such as p.L483P (6.4–30.4), intermediate for mild variants such as p.N409S (2.2–7.8), and lower for risk variants such as p.E365K (1.6–5.5)^1,6^. Several case-control studies have reported that PD patients carrying severe *GBA1* variants compared to carriers of mild or risk variants, present with a more severe clinical phenotype characterised by faster progression, and worse psychiatric and cognitive dysfunction, worsened by interventions such as DBS^4,7–10^. However, recent studies have contradicted these results, reporting similar cognitive and motor deterioration in *GBA1* variant carriers, regardless of variant type^11–14^. Phase II trials are now underway evaluating changes in cognitive function at 12 months as primary outcomes, recruiting either mild or severe variant *GBA1*-PD^15^. An understanding of the natural clinical history of PD associated with different *GBA1* variants is crucial to appropriate trial design.

Here, we present our experience from a large, multi-centre, international cross-sectional study incorporating PD patients negative (idiopathic PD-iPD) or positive for *GBA1* variants (*GBA1*-PD), the latter group being representative of all variant types (risk, mild, and severe). We highlight how the severe *GBA1* variant carriers represent the only group with a remarkably different and more severe clinical phenotype compared to iPD. Our findings caution the PD community to avoid generalisation and consider *GBA1*-PD as a clinically heterogeneous condition.

Demographic and descriptive features of iPD and *GBA1*-PD groups are summarised in Table 1, while clinical features are summarised in Table 2. A total of 301 PD patients (183 iPD, 118 *GBA1*-PD) were recruited. *GBA1* variants were classified into three classes (severe, mild, and risk) as previously reported (a list of *GBA1* variants included in the study is presented in Supplementary Table 1)^6^. Within the *GBA1*-PD, 47% (*N* = 55) carried *GBA1* risk variants, 33% (*N* = 39) severe variants, and 20% (*N* = 24) mild variants.

**Table 1:** Demographics and descriptive features of study cohort

	Groups		*p value*	Groups			*p value*^a^					
	iPD (*N* = 183)	*GBA1*-PD (*N* = 118)	iPD vs *GBA1*-PD	Risk (*N* = 55)	Mild (*N* = 24)	Severe (*N* = 39)	iPD vs Risk	iPD vs Mild	iPD vs Severe	Risk vs Mild	Risk vs Severe	Mild vs Severe
**GBR (N)**	89	60	-	35	12	13	-	-	-	-	-	-
**ITA (N)**	94	58	-	20	12	26	-	-	-	-	-	-
**Age**	65 ± 9.0	60 ± 9.3	<0.0001	63 ± 9	57 ± 10	58 ± 9	ns	0.0001^*^	<0.0001^*^	0.0053^*^	0.0111^*^	ns
**Sex (% F, N)**	42% (77)	46% (54)	ns	36% (20)	54% (13)	54% (21)	ns	ns	ns	ns	ns	ns
**PD family history (%, N)**	23% (42)	35% (41)	0.0354	33% (18)	25% (6)	44% (17)	ns	ns	0.0143	ns	ns	ns
**Education (yrs)**	13.8 ± 4.4	14.3 ± 4.2	ns	14.3 ± 4.6	14.1 ± 3.4	14.3 ± 4.1	ns	ns	ns	ns	ns	ns
**BMI (kg/m**^2^**)**	25.8 ± 5.2	25.5 ± 4.5	ns	26 ± 5	24.4 ± 3.4	25.5 ± 4.5	ns	ns	ns	ns	ns	ns
**PD Age at onset**	59 ± 9.2	54 ± 10.0	<0.0001	58 ± 8.9	50 ± 10.9	52 ± 9.5	ns	<0.0001^*^	<0.0001^*^	0.0014^*^	0.0039^*^	ns
**PD Duration**	6.1 ± 5.1	6.5 ± 4.5	ns	6 ± 3.7	7.2 ± 5.8	6.8 ± 4.6	ns	ns	ns	ns	ns	ns
**H&Y stage (median)**	2	2	ns	2	2	2	ns	ns	ns	ns	ns	ns
**LEDD (mg)**	559 ± 430.2	659.5 ± 488.5	ns	611.8 ± 532.9	655.3 ± 474.5	729.5 ± 432.1	ns	ns	ns	ns	ns	ns
**DBS (%, N)**	8% (15)	17.8% (21)	0.0201	9% (5)	12.5% (3)	33% (13)	ns	ns	0.0001^*^	ns	0.0074^*^	ns
**DBS Target (%, N)**^#^	27% (4) GPi, 73% (11) STN	19% (4) GPi, 81% (17) STN	ns	40% (2) GPi, 60% (3) STN	100% (3) STN	15% (2) GPi, 85% (11) STN	ns	ns	ns	ns	ns	ns
**Apomorphine pump infusion (N, %)**	-	0.8% (1)	-	-	4% (1)	-	-	-	-	-	-	-
**LCIG (N, %)**	-	0.8% (1)	-	-	4% (1)	-	-	-	-	-	-	-

Data are presented as mean ± sd, unless otherwise stated.

^#^Percentages were calculated on the basis of the number of participants who had DBS surgery.

^a^unadjusted *p* values indicating differences among iPD, risk, mild and severe *GBA1*-PD.

^*^significant *p* values that resisted to adjustment for multiple group comparisons (iPD, risk, mild, severe *GBA1*-PD).

*BMI* body mass index, *DBS* deep brain stimulation, *GPi* globus pallidus internus, *H&Y* Hoehn & Yahr, *LCIG* levodopa-carbidopa intestinal gel, *GBR* Great Britain, *ITA* Italy, *NA* not available, *ns* non-significant, *PD* Parkinson disease, *STN* subthalamic nucleus.

**Table 2:** Clinical features of study cohort

	Groups		*p value*	Groups			*p value*^*a*^					
	iPD (*N* = 183)	*GBA1*-PD (*N* = 118)	iPD vs *GBA1*-PD	Risk (*N* = 55)	Mild (*N* = 24)	Severe (*N* = 39)	iPD vs Risk	iPD vs Mild	iPD vs Severe	Risk vs Mild	Risk vs Severe	Mild vs Severe
**MDS-UPDRS part I**	9.9 ± 6.9	10.8 ± 6.2	ns	10.2 ± 6.5	10.4 ± 5.5	11.8 ± 6.1	ns	ns	ns	ns	ns	ns
**MDS-UPDRS part II**	10.4 ± 7	11.4 ± 6.1	ns	11.1 ± 6.1	10.6 ± 6.3	12.3 ± 6.1	ns	ns	ns	ns	ns	ns
**MDS-UPDRS part III**	27 ± 12.8	27.8 ± 12.9	ns	28.1 ± 12.5	26.9 ± 12.1	27.9 ± 14.2	ns	ns	ns	ns	ns	ns
**MDS-UPDRS part IV**	2.6 ± 3.4	3.7 ± 3.6	0.0399	3.4 ± 3.3	3.7 ± 4	4.2 ± 3.8	ns	ns	ns	ns	ns	ns
**MDS-UPDRS total**	49.5 ± 23	53.7 ± 20.2	ns	52.8 ± 19.7	51.6 ± 18.7	56.2 ± 21.8	ns	ns	ns	ns	ns	ns
**SCOPA-AUT total**	13.7 ± 7.4	14.3 ± 7.1	ns	13.5 ± 6.9	14.2 ± 7.9	15.4 ± 7.1	ns	ns	ns	ns	ns	ns
**WCSS**	5.8 ± 4.4	6.1 ± 4.5	ns	5.3 ± 3.8	6.1 ± 4.8	7.2 ± 5.1	ns	ns	0.0305	ns	0.0322	ns
**RBD-positive (%, N)**	37% (67)	43% (50)	ns	44% (24)	43% (10)	41% (16)	ns	ns	ns	ns	ns	ns
**UPSIT**	18.8 ± 6.7	17.4 ± 5.5	0.0054	17.5 ± 5.1	18.9 ± 5.2	16.4 ± 6.2	ns	ns	0.0016^*^	ns	ns	ns
**BDI**	8.7 ± 6.6	11.3 ± 6.4	0.0026	10.1 ± 5.9	12.8 ± 8.8	12.2 ± 5.2	ns	0.0139^*^	0.0101^*^	ns	ns	ns
**MOCA**	25.7 ± 3.5	26.0 ± 3.8	ns	26.3 ± 3.6	27.2 ± 2.5	24.9 ± 4.4	ns	ns	0.0296	ns	0.0117^*^	0.0114^*^

Data are presented as mean ± sd, unless otherwise stated.

^a^unadjusted *p* values indicating differences among iPD, risk, mild and severe *GBA1*-PD.

^*^significant *p* values that resisted to adjustment for multiple group comparisons (iPD, risk, mild, severe *GBA1*-PD).

*BDI* Beck Depression Inventory, *LEDD* levodopa equivalent daily dose, *MDS-UPDRS* Movement Disorder Society (MDS) Unified Parkinson’s Disease Rating Scale, *MOCA*, Montreal Cognitive Assessment, *ns* non-significant, *PD* Parkinson disease, *SCOPA-AUT* SCales for Outcomes in PArkinson’s disease, *RBD* REM Sleep Behaviour Disorder, *UPSIT* University of Pennsylvania Smell Identification Test, *WCSS* Wexner Constipation Scoring System.

Compared with iPD, *GBA1*-PD patients showed a significantly younger age (60 vs 65 years) and more frequent positive family history for PD (35% vs 23% of participants). No differences in sex distribution were found between groups. In terms of disease-associated features, *GBA1*-PD patients developed the disease 5 years earlier and had undergone DBS more frequently than iPD patients. No differences in disease duration or dopaminergic medication doses (calculated as levodopa equivalent daily dose-LEDD) were identified. *GBA1*-PD patients presented with more severe depression (β = 2.3, *p* = 0.0026), olfactory dysfunction (β = −2.03, *p* = 0.0054), and motor complications (β = 0.8, *p* = 0.0399). Although no differences emerged in global cognitive and autonomic function, in an exploratory analysis, individual items of the MOCA test and composite items assessing the 6 sub-domains of the SCOPA-AUT scale were compared between groups (Supplementary Tables 2 and 3). The *GBA1*-PD group showed only a trend for worse performances in tasks evaluating executive/visuospatial functions and greater cardiovascular dysfunction.

When patients were stratified according to *GBA1* variant type, severe and mild *GBA1*-PD were significantly younger than iPD and risk *GBA1*-PD, with no difference in age between severe and mild *GBA1*-PD. Severe and mild *GBA1*-PD also developed PD earlier than both iPD and risk *GBA1*-PD. Only severe *GBA1*-PD patients were more frequently subjected to DBS procedure compared to iPD patients and tended to have a more frequent positive family history for PD (44%) compared to iPD (Table 1). Groups were otherwise homogenous in terms of disease duration and LEDD. When clinical features were compared, severe *GBA1*-PD patients, but not risk or mild *GBA1*-PD patients, presented with a distinct clinical profile when compared to iPD patients (Table 2). This profile was characterised by more severe depression (β = 2.9, *p* = 0.0101) and olfactory dysfunction (β = −3.4, *p* = 0.0016). Global cognitive performances were significantly worse in severe *GBA1*-PD compared with iPD-but this did not resist to adjustment for multiple group comparison (adj.p = 0.059), and compared with risk and mild *GBA1*-PD. More severe constipation was also observed in severe *GBA1*-PD compared with iPD and risk *GBA1*-PD, but this did not resist to multiple group comparison. Compared with iPD, worse depression was the only differing feature found in mild *GBA1*-PD (β = 3.5, *p* = 0.0139), whereas no differing features were observed between iPD and risk *GBA1*-PD. When we investigated specific items of the MOCA and SCOPA-AUT scales within the subgroups of *GBA1* variant carriers, we identified that visuospatial abilities and pupillomotor function were mainly affected in severe *GBA1*-PD compared with iPD and risk *GBA1*-PD (Supplementary Tables 2 and 4). Results on cognitive outcomes showed even more significant differences between iPD and severe *GBA1*-PD when analyses were repeated excluding patients with DBS (*N* = 36), suggesting that genetic *GBA1* status itself plays an independent role in cognitive decline (Supplementary Tables 5 and 6).

In this large, multi-centre, international cross-sectional study, we demonstrated the importance of considering the type of *GBA1* genetic variant in research studies and clinical trials conducted on *GBA1*-PD. Reporting findings on *GBA1*-PD without stratifying according to *GBA1* variant type, can be misleading: differences can be detected related to iPD, however these are mainly driven by severe variant carriers.

Our results align with previous findings showing a more severe phenotype in severe variant carriers^8,10,16^. In a previous study comparing a large cohort of *GBA1*-PD (139 mild, 48 severe) to 152 iPD patients, more severe depression, hallucinations, worse hyposmia, and higher frequencies of REM sleep behaviour disorder (RBD) were detected in severe *GBA1*-PD compared to the other two groups^16^. However, this study did not include risk variant carriers, who numerically represent the predominant group of *GBA1*-PD individuals, especially in those of Caucasian background^1^.

In contrast to previous reports, we did not detect any significant difference in cognitive impairment in carriers of risk or mild variants compared to iPD. One study found that both pathogenic (mix of mild and severe) variant carriers (*N* = 60) and p.E365K carriers (*N* = 65) had higher incidence of dementia and a greater impairment in working memory/executive function and visuospatial abilities compared to iPD, however the disease duration in these groups was mostly >7.5years, so additional factors beyond *GBA1* status might have contributed to these results^13^. Cognitive decline at 7 years from diagnosis was faster in pathogenic and risk variant carriers than iPD in another study, however, the association with progression rate to dementia was much smaller in the risk variant carriers group, and no distinction was made between severe and mild variant carriers^17^.

Our results showed a trend for more severe constipation in severe *GBA1*-PD compared to iPD and risk *GBA1*-PD. Higher burden of gastrointestinal disturbances is a feature of the ‘body-first’ subtype of PD, reflecting more widespread damage to the autonomic nervous system, and it has been hypothesised that individuals with *GBA1* variants often display a phenotype resembling body-first PD^18^. In addition, in our study, we observed higher scores in the SCOPA pupillomotor subdomain in severe *GBA1*-PD, which might be related to a more extreme pupillary parasympathetic dysfunction in this group. Interestingly, pupillomotor abnormalities suggestive of dysfunctional parasympathetic innervation were also detected in individuals with neuronopathic Gaucher disease, caused by biallelic pathogenic variants in *GBA1*^19^, or with Fabry disease, another lysosomal storage disorder^20^. Whether a higher burden of autonomic dysfunction, reflecting more severe impairment of the autonomic nervous system in line with the ‘body-first’ PD subtype, is a distinctive and unique trait of carriers of severe *GBA1* variants, is of great interest to help understand the pathogenesis of *GBA1*-PD.

Several disease-modifying therapies targeting the *GBA1* pathway are in Phase II/III of clinical trial stage, aiming to halt/slow *GBA1*-PD progression^5,21^. Recently, a Phase II, randomized-controlled trial (MOVES-PD), testing the effect of the glucosylceramide synthase inhibitor Venglustat in *GBA1*-PD patients, did not meet its primary endpoint, and showed paradoxical worsening of motor symptoms in mild *GBA1*-PD treated with Venglustat^22^. This, once again, highlights that different pathogenesis might underlie different syndromes according to *GBA1* variant, hence different subgroups of *GBA1*-PD patients might respond to different treatments. Other than the stratification of patients based on variant severity, our data suggest that greater success for clinical trials might depend on the choice of clinical outcome measures. Change in cognitive function has been adopted by a recent Phase II clinical trial^15^, but if different trajectories in cognitive decline are primarily driven by *GBA1* status, the effect of disease-modifying therapies on cognition might be confounded.

Our study has some limitations. First, the relatively small number of individuals with severe and mild *GBA1*-PD might have undermined the results. Second, this is a cross-sectional study; however, a 2-year follow-up assessment of this cohort is in progress. Third, our study recruited participants from the United Kingdom and Italy, so *GBA1* variants, which are common in different geographical locations, might have been missed. Fourth, although our analyses were adjusted based on group count, they did not account for the number of tests performed (i.e., clinical features analysed). This approach might increase type I errors; however, our goal was to explore a wide range of clinical measurements including scales never used before in *GBA1*-PD (e.g., evaluating constipation), to gain novel insight on pathophysiology and provide new tentative findings that future studies conducted on larger cohorts could corroborate.

Despite these limitations, we believe that this study provides clinicians with useful information to approach their *GBA1*-PD patients in the clinic, tailoring their counselling on prognosis and disease trajectories, and integrating our recent consensus guidance on PD risk^1^. Moreover, we hope that these findings will inform researchers involved in clinical trials to improve the design of such studies and guide the choice of realistic outcomes in the correct patient groups. Finally, this study provides insight into the pathophysiological mechanisms underlying disease severity in *GBA1*-PD.

## Methods

### Study design and clinical data

Participants were recruited via the RAPSODI study (University College London) in the United Kingdom^23^, and two neurology tertiary centres in Italy (IRCCS Mondino Foundation, Pavia, and Azienda USL-IRCCS, Reggio Emilia). This study was approved by the local Ethics Committees (London – Queen Square REC: 15/LO/1155; EC of Pavia: code P-20210009687; EC of Area Vasta Emilia Nord: code 2021/0092531). All participants signed informed consent upon enrolment.

### *GBA1* sequencing

For the UK cohort, full *GBA1* gene sequencing was performed on saliva samples collected with the DNA OG-500 kit from DNA Genotek and posted back by participants. DNA extraction and long range sequencing of an 8.9 kb amplicon, including all coding exons and introns of *GBA1*, was performed using bespoke primers on the Oxford Nanopore MinION platform, as previously described^24^. This was done partially at the laboratories of the Department of Clinical and Movement Neurosciences, UCL Institute of Neurology, United Kingdom, and partially at the Exeter Clinical Laboratory International, United Kingdom, a diagnostic clinical laboratory accredited by the UK’s National Accreditation Body (UKAS 8092). *GBA1* positive results were confirmed by Sanger sequencing at the Exeter Clinical Laboratory International. The UK participants were also screened for the *LRRK2* G2019S variant, as an exclusion criterion. The *LRRK2* gene was genotyped with KBiosciences Competitive AlleleSpecific PCR SNP genotyping system at the LGC Genomics, Hoddesdon, Herts. Analysis of the *GBA1* gene in the Mondino Pavia cohort was performed by a next-generation sequencing (NGS)-based method, which relies on the selective amplification of the whole *GBA1* gene in one long PCR fragment (6 kb) followed by Nextera sequencing and a customized bioinformatics pipeline aimed at masking the *GBAP1* pseudogene^25^. Identified variants were validated by conventional Sanger sequencing through *GBA1* amplification in three overlapping fragments using specific primer pairs. Pathogenic variants in 15 PD-related genes associated with autosomal dominant (*SNCA*, *LRRK2*, *VPS35*, *GBA1*), X-linked (*RAB39B*), and autosomal recessive PD (*PRKN*, *PINK1*, *PARK7*, *ATP13A2*, *PLA2G6*, *DNAJC6*, *SYNJ1*, *FBXO7*, *VPS13C*, *PTRHD1*) were searched for by means of NGS-based sequencing of Parkinson disease (PD) gene virtual panel as well as MLPA analysis (SALSA Kit P51-P52, MRC Holland). In the Reggio Emilia cohort, analysis of the *GBA1* gene for PD patients was performed by combining a primary *GBA1*-specific long-range PCR with subsequent *GBA1* exon-specific PCR and NGS of the resulting products, as previously described^2^. Patients were also tested for 11 pathogenic or likely pathogenic *LRKK2* variants. If negative, 50 target genes were analysed by means of NGS-based sequencing of the PD gene virtual panel^2^. For non-Parkinsonian individuals of the Reggio Emilia cohort, the genetic analysis of the *GBA1* gene was performed either by a long PCR approach or NGS-based method, as previously described^26,27^.

### Clinical evaluation

Demographic information was collected from each participant enroled in the study. Information about family history, disease onset, and medications was collected, and levodopa equivalent daily dose (LEDD) calculated for each participant^28,29^. An extensive range of clinical scales and questionnaires was administered to each participant, to evaluate motor and non-motor (autonomic function, RBD, olfaction, mood, cognition) symptoms of PD. These included: Movement Disorder Society-Unified Parkinson’s Disease Rating Scale (MDS-UPDRS, part I-IV)^30^, Hoehn & Yahr (H&Y) scale^31^, SCales for Outcomes in PArkinson’s disease (SCOPA-AUT)^32^, Wexner Constipation Scoring System (WCSS)^33^, REM Sleep Behaviour Disorder Questionnaire (RBDSQ)^34^, University of Pennsylvania Smell Identification Test (UPSIT)^35^, Beck Depression Inventory (BDI)^36^, and Montreal Cognitive Assessment (MOCA)^37^.

### Statistical analysis

The first set of analyses compared the iPD group to the *GBA1*-PD group, to highlight clinical features differentiating the two. Subsequently, based on the hypothesis that differences in clinical phenotype between iPD and *GBA1*-PD patients are driven by variant type, patients were stratified according to their *GBA1* variant severity into risk, mild, and severe. Study data were collected and managed using REDCap electronic data capture tools^38^. Categorical variables were compared using χ2 test or Fisher’s exact test. Continuous variables are presented as means ± SDs, unless otherwise specified. Group demographics and descriptive features were compared using t-test or non-parametric Wilcoxon rank-sum test for two-group comparison (iPD vs *GBA1*-PD), or One-way ANOVA or non-parametric Kruskal-Wallis test with multiple pairwise t-tests or the non-parametric analogue Dunn’s pairwise tests for multiple-group comparisons (iPD vs risk *GBA1*-PD vs mild *GBA1*-PD vs severe *GBA1*-PD). Differences within clinical variables between groups were analysed using linear regression models for continuous variables, including age, sex and disease duration as co-variates. For global MOCA scores, scores were initially adjusted for education level according to instructions^37^. For RBDSQ scores, data were categorised using clinically validated thresholds and binary regression was used to determine associations between categorised variables and the grouping variable, and analysis was adjusted for age, sex and disease duration as co-variates. Individual sub-scores of the MOCA scale were analysed using ordinal logistic regression (OLR) or binary regression according to the outcome, with analysis adjusted for the same co-variates and for years of education. Correction of *p*-values using the Benjamini-Hochberg (BH) procedure was applied to multiple-group comparisons (iPD vs risk *GBA1*-PD vs mild *GBA1*-PD vs severe *GBA1*-PD). Statistical analyses were performed using R version 4.2.3^39^.

## Supplementary information

Supplementary material

## Data Availability

Data used in the preparation of this article were deposited to the Global Parkinson’s Genetics Program (GP2; https://gp2.org) as Tier 2 data. They are part of GP2 release 11 (10.5281/zenodo.7904831), which is planned for release in December 2025. GP2 data are available on AMP PD (https://amp-pd.org). The code used to generate the results of this article and the Key Resource Table can be found at https://zenodo.org/records/15781438.
