# Supplementary material for: Severe GBA1 variants drive the GBA1-PD clinical phenotype: implications for counselling and clinical trials
Source: NPJ Parkinsons Dis. 2025 Oct 1;11:281. doi: 10.1038/s41531-025-01063-3 (PMC12488979; doi:10.1038/s41531-025-01063-3)
Supplement: Supplementary file 1 — Supplementary material [file 41531_2025_1063_MOESM1_ESM.docx]

**Supplementary material**

**Severe *GBA1* variants drive the *GBA1*-PD clinical phenotype: implications for counselling and clinical trials**

**Table of Contents:**

- **Supplementary Tables (p. 2-7)**
- **References (p. 8)**

**Supplementary Table 1. List of *GBA1* variants.**

| **Genotype** | **N** | **Variant severity** |
| --- | --- | --- |
| G241R/WT | 2 | severe |
| H294Q+D448H/WT | 1 | severe |
| IVS2+1G>A/WT | 1 | severe |
| K(-27)R+L483P+P491P/WT | 1 | severe |
| L483P/WT | 18 | severe |
| N227S/WT | 3 | severe |
| P284T/WT | 1 | severe |
| Q239Ter/WT | 1 | severe |
| Q401Ter/WT | 1 | severe |
| R170C/WT | 1 | severe |
| R202Ter/WT | 1 | severe |
| R296Q/WT | 1 | severe |
| R296Ter/WT | 1 | severe |
| R502C/WT | 4 | severe |
| RecNcil/WT | 1 | severe |
| V433L/WT | 1 | severe |
|  |  |  |
| N409S/WT | 22 | mild |
| R209P/WT | 1 | mild |
| R368C/WT | 1 | mild |
|  |  |  |
| E365K/WT | 33 | risk |
| T408M/E365K | 2 | risk |
| T408M/WT | 20 | risk |

Variants were classified according to the *GBA1*-PD browser^1^ (except for variants P284T classified as severe because it was previously associated with absent GCase activity^2^, and Q401Ter classified as severe because it results in a premature truncation of the protein).

**Supplementary Table 2. Sub-domains of the SCOPA-AUT scale.**

|  | **Groups** | | ***p value*** | **Groups** | | | ***p value ^a^*** | | | | | |
| --- | --- | --- | --- | --- | --- | --- | --- | --- | --- | --- | --- | --- |
|  | **iPD (N=183)** | ***GBA1*-PD**  **(N=118)** | **iPD vs *GBA1*-PD** | **Risk**  **(N=55)** | **Mild**  **(N=24)** | **Severe**  **(N=39)** | **iPD vs Risk** | **iPD vs Mild** | **iPD vs Severe** | **Risk vs Mild** | **Risk vs Severe** | **Mild vs Severe** |
| **SCOPA-AUT Gastrointestinal** | 3.8 ± 2.9 | 4.1 ± 2.8 | ns | 3.9 ± 2.6 | 3.7 ± 2.8 | 4.6 ± 3 | ns | ns | ns | ns | ns | ns |
| **SCOPA-AUT Urinary** | 5.1 ± 3.1 | 4.8 ± 2.9 | ns | 5 ± 3.2 | 4.2 ± 2.7 | 4.9 ± 2.5 | ns | ns | ns | ns | ns | ns |
| **SCOPA-AUT Cardiovascular** | 0.7 ± 1 | 0.9 ± 1.1 | ns *(p=0.0907)* | 0.8 ± 1 | 1 ± 1.2 | 1 ± 1.2 | ns | ns | ns | ns | ns | ns |
| **SCOPA-AUT Thermoregulatory** | 2.1 ± 1.9 | 2.5 ± 2.4 | ns | 2.3 ± 2.2 | 3.1 ± 3 | 2.5 ± 2.3 | ns | ns | ns | ns | ns | ns |
| **SCOPA-AUT Pupillomotor** | 0.5 ± 0.7 | 0.5 ± 0.9 | ns | 0.4 ± 0.8 | 0.5 ± 0.9 | 0.7 ± 1 | ns | ns | 0.0507 | ns | 0.0467 | ns |
| **SCOPA-AUT Sexual** | 1.5 ± 1.8 | 1.4 ± 1.7 | ns | 1.1 ± 1.7 | 1.8 ± 1.9 | 1.6 ± 1.6 | ns | ns | ns | 0.0417 | 0.0488 | ns |

Legend: ns: not significant; PD, Parkinson disease.

^a^: unadjusted p values indicating differences among iPD, risk, mild and severe *GBA1*-PD.

^*^: significant p values that resisted to adjustment for multiple comparisons across the 4 groups (iPD, risk, mild, severe *GBA1*-PD).

**Supplementary Table 3. Performances in sub-scores of MOCA test within iPD and *GBA1*-PD.**

|  | **Groups** | | ***p value*** |
| --- | --- | --- | --- |
| **MOCA sub-scores** | **iPD (N=183)** | ***GBA1*-PD (N=118)** | **iPD vs *GBA1*-PD** |
| **Diagram** | 0.9 ± 0.4 | 0.8 ± 0.4 | ns (*p=0.0937*) |
| **Cube** | 0.7 ± 0.4 | 0.7 ± 0.5 | ns (*p=0.0641*) |
| **Clock** | 2.6 ± 0.7 | 2.5 ± 0.8 | ns |
| **Naming** | 2.9 ± 0.3 | 2.9 ± 0.5 | ns |
| **Digits** | 1.8 ± 0.5 | 1.8 ± 0.4 | ns |
| **Letters** | 0.9 ± 0.3 | 0.9 ± 0.3 | ns |
| **Subtraction** | 2.7 ± 0.6 | 2.7 ± 0.7 | ns |
| **Repeat** | 1.7 ± 0.5 | 1.8 ± 0.5 | ns |
| **Fluency** | 0.8 ± 0.4 | 0.8 ± 0.4 | ns |
| **Abstraction** | 1.7 ± 0.5 | 1.7 ± 0.5 | ns |
| **Delayed recall** | 2.8 ± 1.7 | 3.3 ± 1.5 | ns |
| **Orientation** | 5.9 ± 0.4 | 5.8 ± 0.5 | ns |

Legend: CI: confidence interval; ns: not significant; OR: odds ratio; PD, Parkinson disease.

**Supplementary Table 4. Performances in sub-scores of the MOCA test within *GBA1*-PD subgroups (only group comparisons with statistically significant results are reported).**

|  | **Groups** | | | | ***p value*** | **OR (95% CI)** | ***p value*** | **OR (95% CI)** | ***p value*** | **OR (95% CI)** |
| --- | --- | --- | --- | --- | --- | --- | --- | --- | --- | --- |
|  | **iPD (N=183)** | **Risk**  **(N=55)** | **Mild**  **(N=24)** | **Severe**  **(N=39)** | **iPD vs Mild** | | **iPD vs Severe** | | **Risk vs Severe** | |
| **Diagram** | 0.9 ± 0.4 | 0.9 ± 0.4 | 0.8 ± 0.4 | 0.8 ± 0.4 | ns | ns | ns | ns | ns | ns |
| **Cube** | 0.7 ± 0.4 | 0.8 ± 0.4 | 0.8 ± 0.4 | 0.6 ± 0.5 | ns | ns | 0.0022^*^ | 0.2 (0.1-0.6) | 0.0193 | 0.3 (0.1-0.8) |
| **Clock** | 2.6 ± 0.7 | 2.5 ± 0.8 | 2.6 ± 0.6 | 2.4 ± 0.8 | ns | ns | 0.0369 | 0.4 (0.2-0.9) | ns | ns |
| **Naming** | 2.9 ± 0.3 | 2.9 ± 0.4 | 2.9 ± 0.3 | 2.8 ± 0.6 | ns | ns | ns | ns | ns | ns |
| **Digits** | 1.8 ± 0.5 | 1.8 ± 0.4 | 1.9 ± 0.3 | 1.8 ± 0.5 | ns | ns | ns | ns | ns | ns |
| **Letters** | 0.9 ± 0.3 | 1 ± 0.2 | 1 ± 0.2 | 0.8 ± 0.4 | ns | ns | ns | ns | ns | ns |
| **Subtraction** | 2.7 ± 0.6 | 2.8 ± 0.5 | 2.9 ± 0.3 | 2.4 ± 0.9 | ns | ns | ns | ns | 0.0136 | 0.3 (0.09-0.8) |
| **Repeat** | 1.7 ± 0.5 | 1.7 ± 0.5 | 1.8 ± 0.4 | 1.8 ± 0.4 | ns | ns | ns | ns | ns | ns |
| **Fluency** | 0.8 ± 0.4 | 0.7 ± 0.4 | 0.9 ± 0.3 | 0.7 ± 0.4 | ns | ns | ns | ns | ns | ns |
| **Abstraction** | 1.7 ± 0.5 | 1.8 ± 0.5 | 1.8 ± 0.4 | 1.6 ± 0.6 | ns | ns | ns | ns | ns | ns |
| **Delayed recall** | 2.8 ± 1.7 | 3.3 ± 1.5 | 3.8 ± 1.2 | 3 ± 1.5 | ns | ns | ns | ns | ns | ns |
| **Orientation** | 5.9 ± 0.4 | 5.9 ± 0.5 | 5.9 ± 0.3 | 5.7 ± 0.7 | ns | ns | 0.0333 | 0.3 (0.1-0.9) | ns | ns |

Legend: CI: confidence interval; ns: not significant; OR: odds ratio; PD, Parkinson disease.

^*^: significant p values that resisted to adjustment for multiple comparisons across the 4 groups (iPD, risk, mild, severe *GBA1*-PD).

**Supplementary Table 5. Performances of cognitive outcomes within iPD and *GBA1*-PD after exclusion of N=36 patients who underwent DBS (N=265 in total).**

|  | **Groups** | | ***p value*** | **OR (95% CI)** |
| --- | --- | --- | --- | --- |
|  | **iPD (N=168)** | ***GBA1*-PD (N=97)** | **iPD vs *GBA1*-PD** | |
| **MOCA (total score)** | 26.0 ± 3.2 | 26.2 ± 3.6 | ns | NA |
| **MOCA sub-scores** | | | |  |
| **Diagram** | 0.9 ± 0.3 | 0.8 ± 0.4 | 0.0171 | 0.3 (0.1-0.8) |
| **Cube** | 0.8 ± 0.4 | 0.7 ± 0.4 | ns (*p=0.0559*) | ns |
| **Clock** | 2.6 ± 0.7 | 2.5 ± 0.8 | ns *(p=0.0861)* | ns |
| **Naming** | 3 ± 0.2 | 2.9 ± 0.4 | ns | ns |
| **Digits** | 1.8 ± 0.5 | 1.8 ± 0.4 | ns | ns |
| **Letters** | 0.9 ± 0.3 | 0.9 ± 0.3 | ns | ns |
| **Subtraction** | 2.7 ± 0.5 | 2.7 ± 0.7 | ns | ns |
| **Repeat** | 1.7 ± 0.5 | 1.8 ± 0.5 | ns | ns |
| **Fluency** | 0.8 ± 0.4 | 0.8 ± 0.4 | ns | ns |
| **Abstraction** | 1.8 ± 0.5 | 1.8 ± 0.5 | ns | ns |
| **Delayed recall** | 2.9 ± 1.6 | 3.3 ± 1.4 | ns | ns |
| **Orientation** | 5.9 ± 0.3 | 5.9 ± 0.5 | ns | ns |

Legend: CI: confidence interval; ns: not significant; OR: odds ratio; PD, Parkinson disease.

**Supplementary Table 6. Performances in sub-scores of the MOCA test within *GBA1*-PD subgroups (only group comparisons with statistically significant results are reported), after exclusion of N=36 patients who underwent DBS (N=265 in total).**

|  | **Groups** | | | | ***p value*** | **OR (95% CI)** | ***p value*** | **OR (95% CI)** | ***p value*** | **OR (95% CI)** |
| --- | --- | --- | --- | --- | --- | --- | --- | --- | --- | --- |
|  | **iPD (N=168)** | **Risk**  **(N=50)** | **Mild**  **(N=21)** | **Severe**  **(N=26)** | **iPD vs Mild** | | **iPD vs Severe** | | **Risk vs Severe** | |
| **MOCA (total score)** | 26 ± 3.2 | 26.3 ± 3.6 | 27.0 ± 2.6 | 25.3 ± 4.3 | ns | NA | 0.0304 | NA | 0.0284 | NA |
| **MOCA sub-scores** | | | | | | | | | | |
| **Diagram** | 0.9 ± 0.3 | 0.9 ± 0.4 | 0.8 ± 0.4 | 0.8 ± 0.4 | 0.0084 | 0.2 (0.04-0.7) | 0.0277 | 0.2 (0.05-0.9) | ns | ns |
| **Cube** | 0.8 ± 0.4 | 0.8 ± 0.4 | 0.8 ± 0.4 | 0.6 ± 0.5 | ns | ns | 0.0020^*^ | 0.2 (0.07-0.6) | 0.0153^*^ | 0.2 (0.1-0.8) |
| **Clock** | 2.6 ± 0.7 | 2.5 ± 0.8 | 2.5 ± 0.6 | 2.5 ± 0.8 | ns | ns | 0.0495 | 0.4 (0.2-1.0) | ns | ns |
| **Naming** | 3 ± 0.2 | 2.9 ± 0.5 | 2.9 ± 0.3 | 3 ± 0.2 | ns | ns | ns | ns | ns | ns |
| **Digits** | 1.8 ± 0.5 | 1.8 ± 0.4 | 1.9 ± 0.3 | 1.8 ± 0.5 | ns | ns | ns | ns | ns | ns |
| **Letters** | 0.9 ± 0.3 | 1 ± 0.2 | 0.9 ± 0.2 | 0.8 ± 0.4 | ns | ns | ns | ns | ns | ns |
| **Subtraction** | 2.7 ± 0.5 | 2.8 ± 0.5 | 2.9 ± 0.4 | 2.3 ± 1.0 | ns | ns | 0.0165^*^ | 0.3 (0.12-0.8) | 0.0077^*^ | 0.2 (0.05-0.6) |
| **Repeat** | 1.7 ± 0.5 | 1.8 ± 0.5 | 1.8 ± 0.4 | 1.8 ± 0.4 | ns | ns | ns | ns | ns | ns |
| **Fluency** | 0.8 ± 0.4 | 0.7 ± 0.4 | 0.8 ± 0.4 | 0.8 ± 0.4 | ns | ns | ns | ns | ns | ns |
| **Abstraction** | 1.8 ± 0.5 | 1.8 ± 0.5 | 1.9 ± 0.4 | 1.7 ± 0.6 | ns | ns | ns | ns | ns | ns |
| **Delayed recall** | 2.9 ± 1.6 | 3.2 ± 1.5 | 3.8 ± 1.1 | 3.2 ± 1.5 | ns | ns | ns | ns | ns | ns |
| **Orientation** | 5.9 ± 0.3 | 5.9 ± 0.5 | 5.9 ± 0.3 | 5.7 ± 0.7 | ns | ns | ns | ns | ns | ns |

Legend: CI: confidence interval; ns: not significant; OR: odds ratio; PD, Parkinson disease.

^*^: significant p values that resisted to adjustment for multiple comparisons across the 4 groups (iPD, risk, mild, severe *GBA1*-PD).

**References**

1 Parlar, S. C., Grenn, F. P., Kim, J. J., Baluwendraat, C. & Gan-Or, Z. Classification of GBA1 Variants in Parkinson's Disease: The GBA1-PD Browser. *Mov Disord* **38**, 489-495 (2023). <https://doi.org/10.1002/mds.29314>

2 Malini, E. *et al.* Functional analysis of 11 novel GBA alleles. *Eur J Hum Genet* **22**, 511-516 (2014). <https://doi.org/10.1038/ejhg.2013.182>
